# Virulence and Antibiotic Resistance Profiles of *Cronobacter sakazakii* and *Enterobacter* spp. Involved in the Diarrheic Hemorrhagic Outbreak in Mexico

**DOI:** 10.3389/fmicb.2018.02206

**Published:** 2018-09-27

**Authors:** Julio Parra-Flores, Juan Aguirre, Vijay Juneja, Emily E. Jackson, Ariadnna Cruz-Córdova, Jesus Silva-Sanchez, Stephen Forsythe

**Affiliations:** ^1^Departamento de Nutrición y Salud Pública, Facultad Ciencias de la Salud y de los Alimentos, Universidad del Bío-Bío, Chillán, Chile; ^2^Departamento Agroindustria y Enología, Facultad de Ciencias Agronómicas, Universidad de Chile, Santiago, Chile; ^3^Residue Chemistry and Predictive Microbiology Research Unit, Eastern Regional Research Center, Agricultural Research Service, United States Department of Agriculture (USDA), Wyndmoor, PA, United States; ^4^Department of Biology, University of Nevada, Reno, Reno, NV, United States; ^5^Laboratorio de Bacteriología Intestinal, Hospital Infantil de México, Federico Gómez, Mexico City, Mexico; ^6^Grupo de Resistencia Bacteriana, Instituto Nacional de Salud Pública, Cuernavaca, Morelos, Mexico; ^7^Foodmicrobe.com, Nottingham, United Kingdom

**Keywords:** *Cronobacter sakazakii*, *Enterobacter hormaechei*, powdered infant formula, virulence, antibiogram

## Abstract

*Cronobacter* spp. are bacterial pathogens that cause neonatal meningitis, septicemia, and necrotizing enterocolitis in infants with a lethality rate of 40–80%. Powdered infant formulas (PIF) have been implicated as the main vehicles of transmission. This pathogen can also cause infection through contaminated expressed breast milk, and it has been recovered from neonatal feeding tubes of neonates not fed reconstituted PIF and milk kitchen areas. This study analyzed antibiotic resistance profiles and the tissue virulence tests of *Cronobacter sakazakii* and *Enterobacter* spp. recovered from PIF, infant fecal matter‘s, and milk kitchen environment involved in a diarrheic hemorrhagic outbreak in 2011 in Mexico. The strains isolated from the outbreak had similar antibiotic resistance profiles and pathogenicity irrespective of isolation site, however, *C. sakazakii* strains isolated from PIF showed significantly higher invasive profiles than *Enterobacter* spp. (*p* = 0.001) and 83% were resistant to more than one antibiotic. The findings of this study can be used to complement existing information to better control *Cronobacter* and *Enterobacter* spp. contamination in PIF production, prevent its transmission, and improve infant food safety.

## Introduction

*Cronobacter* infections are associated with adults and infants (Bowen and Braden, 2006; Holy and Forsythe, 2014; Alsonosi et al., 2015; Forsythe, 2018). Nevertheless, infections of premature neonates are of particular concern are due to their high lethality rate of between 40 and 80% (Joseph and Forsythe, 2012). The clinical manifestation of this pathogen in infants is mainly meningitis, septicemia, and necrotizing enterocolitis (Nazarowec-White and Farber, 1999; Van Acker et al., 2001; Baumbach et al., 2009; Hariri et al., 2013b) although diarrheal and urinary infections have also been observed (Friedemann, 2009).

From 2003 to 2009, 544 cases of *Cronobacter* spp. infection were identified in 6 states of the United States, especially among children <5 years of age (Patrick et al., 2014), indeed, *Cronobacter* is the genus that is the most commonly involved in cases of illness associated mainly with the consumption of contaminated powdered infant formula (PIF) rehydrated (Food Agriculture Organization of the United Nations [FAO] and World Health Organization [WHO], 2004, 2006) although, additional possible reservoirs from preparation utensils and the environment have been recognized (Friedemann, 2008; Siqueira-Santos et al., 2013; Holy and Forsythe, 2014) and contaminated expressed breast milk, where *C. malonaticus* strain was isolated from a breast abscess (Bowen et al., 2017). Additionally, *C. sakazakii* has been isolated from the enteral feeding tubes of neonates not fed reconstituted infant formula (Hurrell et al., 2009).

There are recommended biochemical methods to identify *Cronobacter* spp. (Api20E, ID32E, BIOLOG microarray, Vitek 2 System), but these can only be used for presumptive identification and they can have accuracy level as low as 43% (Cetinkaya et al., 2012; Joseph et al., 2013; Jackson and Forsythe, 2016). Several PCR primers have been proposed to identify members of the genus *Cronobacter* by amplifying specific sequences of variable and conserved regions of the 16S rRNA of the bacteria (Lehner et al., 2004; Hassan et al., 2007). Specific primers for the *rpoB* gene encoding the β region of the polymerase enzyme have been proposed for identifying *Cronobacter* species, but have not taken into account changes in the taxonomy of the species, giving false positive results with some *Enterobacter* species (Jackson et al., 2015; Jackson and Forsythe, 2016).

Baldwin et al. (2009) developed a 7-loci multilocus sequence typing (MLST) scheme for *Cronobacter* speciation and genotyping. The MLST scheme has an open access database^1^ that contains >2,400 strains and >350 whole genomes along with corresponding metadata and updates according to changes in taxonomy. This approach has led to the recognition of clonal complexes (CC) within the *Cronobacter* genus. Of special significance is the recognition of the *Cronobacter sakazakii* CC4 pathovar which is strongly associated with neonatal meningitis cases (Joseph and Forsythe, 2011; Sonbol et al., 2013; Hariri et al., 2013a; Forsythe et al., 2014; Forsythe, 2018).

Jackson et al. (2015) provided the re-evaluation of a previous study done by Flores et al. (2011) of *C. sakazakii* outbreak caused by consuming contaminated reconstituted PIF in Mexico, which had used phenotyping and *rpoB* PCR probe method to identify the isolates, whereas Jackson et al. (2015) used DNA sequencing, and showed that the strains were *E. hormaechei* and *Enterobacter* spp. (undesignated species), demonstrating for the first time, the possible transmission of *Enterobacter* from PIF to infants (Jackson et al., 2015). This possible transmission suggests that these organisms may pose a risk to infants consuming rehydrated PIF (Jackson et al., 2015). In fact, this risk was estimated by Parra-Flores et al. (2016) in a risk based assessment under a probabilistic approach of reconstituted PIF contaminated with different inoculum size of *Cronobacter*, differing heat treatment to prepare the PIF and storage temperature.

Important aspects to be considered in the severity and prognosis of *Cronobacter* infection are the presence of antibiotic-resistance (Caubilla-Barron et al., 2007; Kilonzo-Nthenge et al., 2012; Xu et al., 2015), and virulence factors (Townsend et al., 2008). Such virulence factors can include iron acquisition and the invasiveness and adhesion in cell lines such as HEp-2 and CaCo-2 (Pagotto et al., 2003; Mange et al., 2006; Grim et al., 2012; Almajed and Forsythe, 2016).

The aim of this work was to evaluate and compare the virulence and antibiotic resistance profiles of the *Cronobacter sakazakii* and *Enterobacter* spp. involved in the diarrheic hemorrhagic outbreak in Mexico in 2011.

## Materials and Methods

### Bacterial Strains

**FIGURE 1:**
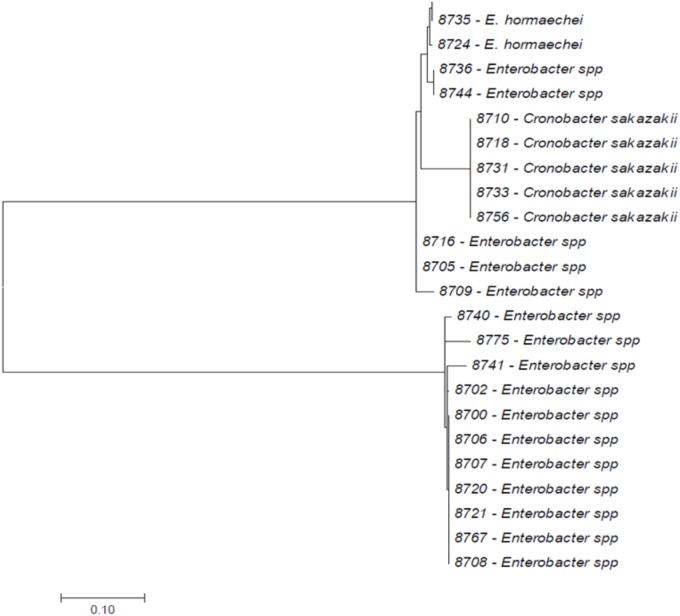
The phylogenetic tree of fusA sequencing of *Cronobacter sakazakii, Enterobacter hormaechei*, and *Enterobacter* spp. The tree with the highest log likelihood (–1450.2262) is shown. Initial tree using the Maximum Composite Likelihood (MCL). The tree is drawn to scale, with branch lengths measured in the number of substitutions per site. The analysis involved 24 nucleotide sequences. Codon positions included were 1st+2nd+3rd+Noncoding. All positions containing gaps and missing data were eliminated. There were a total of 401 positions in the final dataset and analyses were conducted in MEGA7.

All bacterial strains (*n* = 24) had been isolated and identified according to 7-loci MLST as previously described (Jackson et al., 2015) (**Figure 1**). They had been recovered from PIF (*n* = 14), fecal material (*n* = 6), and the PIF preparation area (*n* = 4).

### Sequencing of fusA Gene

The methodology described by Baldwin et al. (2009) was followed using PCR CORE Kit QIAGEN (Cat No. 201225) solutions. Amplified products were sent to MACROGEN in Korea for sequencing. The sequenced products were analyzed with the Gentle software and later aligned with the ClustalW software. A phylogenetic tree was constructed using the maximum likelihood method with the MEGA7 software. Identification was performed with the free access online database https://pubmlst.org/cronobacter/ and BLASTn (NCBI).

### Antibiotic Resistance Profile

The antibiograms of 24 strains were performed by the disk diffusion method (Clinical and Laboratory Standards Institute [CLSI], 2018). Disks with 12 commercial antibiotics were used (Bio-Rad^TM^, United States): ampicillin (10 μg), amikacin (30 μg), levofloxacin (5 μg), cephalothin (30 μg), cefotaxime (30 μg), ceftriaxone (30 μg), chloramphenicol (30 μg), gentamicin (10 μg), netilmicin (30 μg), nitrofurantoin (300 μg), cefepime (30 μg), and sulfamethoxazole-trimethoprim (25 μg). The characterization of the strain resistance/susceptibility profiles was determined by measuring the inhibition area and interpreting the diameters according to the manufacturer’s instructions. *Escherichia coli* ATCC 25922 was used as a reference.

### Virulence Determination of *Cronobacter* and *Enterobacter* spp.

#### Adherence Assay

HEp-2 cells were cultured in Eagle’s minimal medium (MEM) (In Vitro, Mexico) supplemented with 10% fetal bovine serum (FBS) (Gibco, United States) and without antibiotics. The cells were prepared in T75 cm^2^ flasks (Sarstedt, Germany) and grown for 24 h at 37°C and 5% CO_2_. Upon reaching confluency, cells were disaggregated with 0.25% trypsin (In Vitro, Mexico) and sown into 24 well plates from a 1 ml suspension containing 2.5 × 10^6^ HEp-2 cells per ml (Sarstedt, Germany). The monolayers with 70–80% confluency were washed three times with phosphate-buffered saline (PBS) and 900 μl of MEM were added. Isolates were previously cultured overnight in 1% tryptone, and 100 μl bacterial suspensions (10^8^ cells per ml) were added to each well. Plates were incubated for 3 h at 37°C and 5% CO_2_. For quantitative assays, bacteria were removed by adding 1 ml 0.1% Triton X-100 (Amresco, United States), and serial 10-fold dilutions were plated onto tryptone soy agar (TSA) to determine the colony-forming units (CFU) of bacteria bound to HEp-2 cells. Triplicate assays were performed. Enteroaggregative *E. coli* O42 (EAEC) was used as the positive control. *Escherichia coli* K-12 HB101 was the negative control (Cruz et al., 2011). This assay was repeated three times and the results were expressed as the means ± SD of the data.

#### Invasion Assays

The HEp-2 cell lines were prepared according to the procedure described in the adherence assay section. After 3-h incubation, the infected monolayers were washed three times with PBS and incubated with 1 ml MEM with lysozyme 300 μg/ml and gentamycin 100 μg ml^-1^ (In Vitro, Mexico). Samples were washed once with PBS. For quantitative assays, cells were washed three times with PBS, detached with 1 ml 0.1% Triton X-100 and plated on TSA. Invasion frequencies were calculated as the number of bacteria surviving incubation with gentamycin divided by the total number of bacteria present in the absence of this antibiotic (bacterial adherence). Enteroinvasive *E. coli* 1192 and *E. coli* HB101 (K-12) were used as positive and negative controls, respectively. This assay was repeated three times and the results were expressed as the means ± standard deviation of the data (Cruz et al., 2011).

## Results and Discussion

This study is an extension of a previous study by Flores et al. (2011) and Jackson et al. (2015), who suggested that, after the fusA sequence analysis, isolates from the outbreak in Mexico 2011 were a mixture of *C. sakazakii*, *Enterobacter hormaechei*, and *Enterobacter* spp. This was also corroborated by the phylogenetic analysis that clearly showed differences with other strains (**Figure 1**). Currently, the fusA sequencing method enables higher speciation accuracy because it follows the whole genome phylogeny and adjusts to taxonomic changes (Forsythe et al., 2014; Xu et al., 2014; Alsonosi et al., 2015; Jackson et al., 2015).

Several studies have confirmed that *Cronobacter* strains can be effectively eliminated by antibiotics, however, prolonged use of antibiotics, inappropriate dose, quantity and length of application are undesirable as it may result in the development of *Cronobacter* antibiotic resistance (Pérez et al., 2007; Langdon et al., 2016; Fei et al., 2017; Kardos, 2017). Therefore, it is interesting to determine some degree of association in the resistance profiles among strains from food products, environment, and fecal matter of colonized individuals exhibiting or not exhibiting symptoms or from a foodborne outbreak. This aspect is considered part of the objective of the present study because the strains were isolated from PIF, surfaces on which PIF was prepared (**Supplementary Figure S1**), and fecal matter of infants associated to an outbreak occurred in Mexico (Flores et al., 2011). This evaluation enables the design of treatment strategies for exposed individuals, especially those who are hypersensitive such as infants and the elderly. Although, there is considerable speculation about the source of PIF contamination. Some researchers suggest that the sources are either the environment of the production plants (Craven et al., 2010; Jacobs et al., 2011; Fei et al., 2015; Jing-Hua et al., 2015) or the ingredients (i.e., starch) used to prepare the PIF, which are the probable vehicles of transmission (Food Agriculture Organization of the United Nations [FAO] and World Health Organization [WHO], 2004; Jongenburger et al., 2011; Walsh et al., 2011). The Food Agriculture Organization of the United Nations [FAO] and World Health Organization [WHO] (2004, 2006) risk assessments on the microbiological safety of infant formula also recognized that other members of *Enterobacteriaceae* were recoverable from PIF and could put infants at risk even though no confirmed cases had been confirmed at that time.

In our study, the 24 strains were identified as *C. sakazakii* (5), *Enterobacter hormaechei* (3), and *Enterobacter* spp. (16). In general, 83% (20/24) of the isolated strains were resistant to 1–7 antibiotics. Eight percent (2/24) were resistant to 5 antibiotics and 37% (9/24) resistant to 3 antibiotics. Sixty-five percent (16/24) of the resistant strains were isolated from PIF (**Table 1**).

**Table 1:** Resistant antibiotics of isolated strains by source and species.

N° antibiotics tested	Species									N° of resistant strains
	*C. sakazakii*			*E. hormaechei*			*Enterobacter* spp.			
	Source			Source			Source			
	PIF	Surfaces	Fecal	PIF	Surfaces	Fecal	PIF	Surfaces	Fecal	
1	3	–	–	–	–	–	1	–	1	5
2	1	–	–	–	–	–	3	1	–	6
3	–	–	–	1	–	–	3	1	1	6
4	–	–	–	–	–	–	-	–	1	1
5	–	–	–	–	–	1	–	–	–	1
7	–	–	–	–	–	–	–	–	1	1
Total	4	–	–	2	–	1	7	2	4	20

Eighty percent of *C. sakazakii* (4/5) strains were resistant to cephalothin (**Table 2**). It is important to assess the antibiotic resistance profile of *Cronobacter* spp., associated to those product (PIFs and infant products) consumed by high risk population whose are immunologically vulnerable. Molloy et al. (2009) reported that of 33 *C. sakazakii* strains isolated in the environment, 51% were resistant to cephalothin. Kleiman et al. (1981) also reported a moderate resistance to cephalothin in isolated strains in a case of meningoencephalitis.

**Table 2:** Resistant antibiotics profile of *Cronobacter sakazakii*, *E. hormaechei*, and *Enterobacter* spp.

Species	Strains	Source	LEV (5 μg)	FEP (30 μg)	CF (30 μg)	CTX (30 μg)	SXT (25 μg)	AM (10 μg)	CRO (30 μg)	NF (300 μg)	NET (30 μg)	GE (10 μg)	AK (30 μg)	CL (30 μg)
*C. sakazakii*	8710	PIF – México	S	S	R	S	S	S	S	S	S	S	S	S
*C. sakazakii*	8718	PIF – México	S	S	R	S	S	S	S	S	S	S	S	S
*C. sakazakii*	8731	PIF – México	S	S	R	S	S	I	S	S	S	S	S	S
*C. sakazakii*	8733	PIF – México	S	S	R	S	S	R	S	I	S	S	S	S
*C. sakazakii*	8756	Sink washing area (bottles)	S	S	I	S	S	S	S	S	S	S	S	S
*E. hormaechei*	8701	Fecal matter	S	S	R	R	S	R	R	R	S	S	S	S
*E. hormaechei*	8724	PIF – United States	S	S	R	S	S	R	S	R	S	S	S	S
*E. hormaechei*	8735	PIF – México	S	S	R	S	S	R	S	I	S	S	S	S
*Enterobacter* spp.	8700	Fecal matter	S	S	R	R	S	R	R	I	R	R	I	R
*Enterobacter* spp.	8702	Fecal matter	S	S	R	I	S	R	S	R	S	S	S	R
*Enterobacter* spp.	8704	Fecal matter	S	S	S	S	S	S	S	S	S	S	S	S
*Enterobacter* spp.	8705	PIF – United States	S	S	R	I	S	R	S	R	S	S	S	S
*Enterobacter* spp.	8706	Fecal matter	S	S	I	S	S	R	S	R	S	S	S	S
*Enterobacter* spp.	8707	PIF – United States	S	S	S	I	S	S	I	I	S	S	S	S
*Enterobacter* spp.	8708	PIF – United States	S	S	R	I	S	S	I	R	S	S	S	S
*Enterobacter* spp.	8709	Fecal matter	S	S	R	S	S	R	S	R	S	S	S	S
*Enterobacter* spp.	8714	Refrigerator try	S	S	R	S	S	S	S	S	S	S	S	S
*Enterobacter* spp.	8715	Fecal matter	S	R	R	R	S	I	I	S	S	S	S	S
*Enterobacter* spp.	8716	PIF – United States	S	S	R	I	S	S	I	R	S	S	S	S
*Enterobacter* spp.	8717	PIF – United States	S	S	R	S	S	S	S	R	S	S	S	S
*Enterobacter* spp.	8720	PIF – México	S	S	R	S	S	R	S	R	S	S	S	S
*Enterobacter* spp.	8721	PIF – United States	S	S	R	S	S	S	S	R	S	S	S	S
*Enterobacter* spp.	8736	PIF – México	S	S	R	S	S	I	S	S	S	S	S	S
*Enterobacter* spp.	8740	Washing area table	S	S	R	S	S	R	S	R	S	S	S	S
*Enterobacter* spp.	8741	Preparation area handles access	S	R	R	R	S	I	I	S	S	S	S	S
*Enterobacter* spp.	8744	Preparation area table	S	S	S	S	S	S	S	S	S	S	S	S
*Enterobacter* spp.	8766	Sink washing area	S	S	S	S	S	S	S	S	S	S	S	S
*Enterobacter* spp.	8767	Fecal matter	S	S	S	S	S	S	S	R	S	S	S	S
*Enterobacter* spp.	8770	Refrigerator handles	S	S	I	S	S	S	S	S	S	S	S	S
*Enterobacter* spp.	8775	PIF – United States	S	R	I	R	R	R	I	S	S	S	S	S

*LEV Levofloxacin, FEP Cefepime, CF Cephalothin, CTX Cefotaxime, SXT trimethoprim-Sulfamethoxazole, AM Ampicillin, CRO Ceftriaxone, NF Nitrofurantoin, NET Netilmicin, GE Gentamicin, AK Amikacin, CL Chloramphenicol. R: resistant; I: intermediate; S: susceptible.*

For *E. hormaechei*, 100% (3/3) were resistant to cephalothin and ampicillin, 33% (1/3) to cefotaxime and ceftriaxone, and 66% (2/3) to nitrofurantoin. The *Enterobacter* spp. strains were resistant to cephalothin and ampicillin. The resistance values for ampicillin and cephalothin are higher than those previously reported (Kim et al., 2008; Molloy et al., 2009). Lai (2001) reported isolated strains were resistant to the first and second cephalosporin generation. The same situation was found in the present study with the *Cronobacter sakazakii* and *Enterobacter* strains isolated from PIF, milk kitchen surfaces, and fecal matter. Resistance was 26% (4/16) for cefotaxime, 13% (2/16) for ceftriaxone, and 26% (4/16) for cefepime (**Table 2**). This suggests that ß-lactamase production should be further monitored as recommended by the Food Agriculture Organization of the United Nations [FAO] and World Health Organization [WHO], 2008, especially since the resistant strains were isolated from PIF. Caubilla-Barron et al. (2007) analyzed *Cronobacter sakazakii* strains from an outbreak with fatalities in a neonatal intensive care unit in France; they found one pulsotype that was associated with the three fatal cases. These were later shown to be the pathovar *C. sakazakii* CC4 (Joseph and Forsythe, 2011; Masood et al., 2015). In addition, two of these isolates had extended-spectrum ß-lactamase activity. A recent study evaluated the antimicrobial and desiccation resistance of *Cronobacter sakazakii* (Caubilla Barron and Forsythe, 2007), and *Cronobacter malonaticus* isolates from powdered infant formula and processing environments showed that the 70 *Cronobacter* strains, representing 19 sequence types, were susceptible to the most of the antibiotics except for amoxicillin-clavulanate, ampicillin, and cefazolin (Fei et al., 2017) which is in accordance with our results.

**FIGURE 2:**
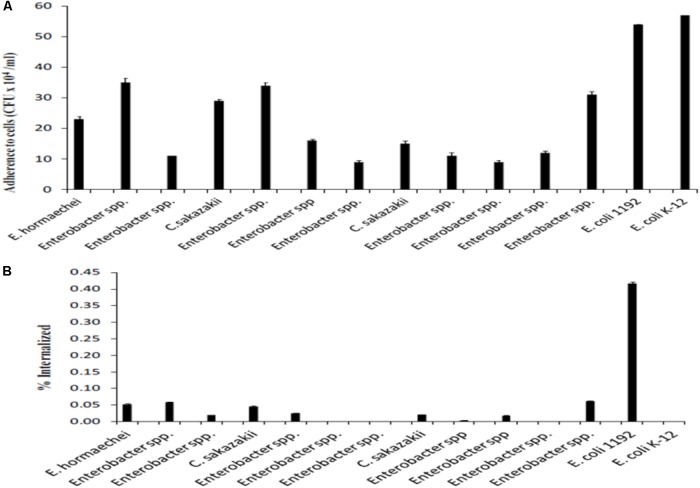
Adherence **(A)** and invasion frequencies **(B)** of the HEp-2 cell line to *C. sakazakii*, *E. hormaechei*, and *Enterobacter* spp.

Our findings indicate that hospitalized infants were unpurposed and accidentally exposed to *Cronobacter* and *Enterobacter* spp. for 2 months. This fact could increase the susceptibility to suffer an infection by this pathogen, especially if this pathogen has a variety of virulence factors which aid in tissue adhesion, invasion and host cell injury. In addition, the results of this study indicate the hospitalized infants were unpurposed and accidentally exposed to *Cronobacter* and *Enterobacter* spp. which were able to adhere and invade human cells (HEp-2 cell line) *in vitro.* This was shown using twelve selected strains which had been isolated from PIF, work surfaces, and fecal matter (**Figure 2**). Due to funding limitations it was impossible to carry out more strains analysis.

Adherence is one of the events that enables bacteria to colonize and invade the host cells; it is a property associated with bacterial pathogenesis, especially of intracellular pathogens (Pizarro-Cerdá and Cossart, 2006; Cruz et al., 2011). In our study, adherence mean values in *Cronobacter* spp., *Enterobacter hormaechei*, and *Enterobacter* spp. HEp-2 cells were 22, 23, and 19 × 10^4^ CFU/mL and with no significant differences among them (*p* > 0.05). Mean invasion values were 3.3, 5.2, and 2.5%, respectively; *E. hormaechei* (*p* = 0.001) was significantly more invasive than *C. sakazakii*. In general, 100% of the evaluated strains had adherence capacity and 75% were invasive in HEp-cells; these values were similar to the results reported by Mange et al. (2006) and Townsend et al. (2008).

Cruz et al. (2011) found five species of *Cronobacter* spp. (*C. sakazakii, C. malonaticus, C. dublinensis, C. muytjensii*, and *C*. genomospecies (current *universalis*), all of which had the capacity to adhere to HEp-2 cell lines. The *C. sakazakii* strains from a human source exhibited higher adherence values compared to strains of the same species isolated from other sources. Furthermore, when the invasion capacity of *C. sakazakii* was evaluated, it was found that 35% of the isolates were invasive and apparently more efficient than the other evaluated *Cronobacter* spp. species.

The *C. sakazakii* and *E. hormaechei* strains evaluated in our study were invasive; however, *Enterobacter* spp. only had 33% of invasive strains, which is of concern because the virulence trait is in isolated PIF strains. Reports of several outbreaks of sepsis in neonatal intensive care units in Brazil and the United States (Campos et al., 2007; Townsend et al., 2008) have shown that *E. hormaechei* is clinically significant, indeed an outbreak of *E. hormaechei* occurred among premature infants in the intensive care nursery (ICN) at the Hospital of the University of Pennsylvania between November 29, 1992 and March 17, 1993 (Wenger et al., 1997).

*Cronobacter* species adhered to HEp-2, Caco-2 and brain microvascular endothelial cells, producing two distinctive adherence patterns, a diffuse and a localized adhesion (Mange et al., 2006; Cruz et al., 2011). Moreover, it has been suggested that the outer membrane proteins OmpA and OmpX from *C. sakazakii* are involved in basolateral invasion of human enterocyte-like Caco-2 and intestinal epithelial cells (Townsend et al., 2007; Singamsetty et al., 2008).

In conclusion, all isolated strains showed resistant to more than one antibiotic (cephalothin, ampicillin, cefotaxime, and ceftriaxone) independent of the source of isolation. In addition, *C. sakazakii* strains isolated from PIF were significantly more invasive than *Enterobacter* spp. Individually; *E. hormaechei* was more invasive than *C. sakazakii* and *Enterobacter* spp.

The knowledge generated in the present work can be used to complement existing information to better control *Cronobacter* and *Enterobacter* spp. contamination in PIF production, prevent its transmission, and improve infant food safety. This information should support regulatory and health authorities in their microbial surveillance measures and improve neonatal and infant health.

## Author Contributions

JP-F conceived the experiments. JP-F, JS-S, AC-C, and JA designed the experiments. JP-F, AC-C, and JS-S conducted the laboratory work. VJ and JA provided data analysis. JP-F, AC-C, JS-S, SF, VJ, and JA drafted the manuscript. EJ revised the manuscript and data analysis. All the authors reviewed and approved the final manuscript.

## Conflict of Interest Statement

The authors declare that the research was conducted in the absence of any commercial or financial relationships that could be construed as a potential conflict of interest.

## Footnotes

1 www.PubMLST.org/cronobacter

## Supplementary Material

FIGURE S1 Milk kitchen floor plan: **(A)** PIF bottles preparation area (sterile), and **(B)** Washing and disinfection area.
