# Supplementary figures and images for: Virulence and Antibiotic Resistance Profiles of Cronobacter sakazakii and Enterobacter spp. Involved in the Diarrheic Hemorrhagic Outbreak in Mexico
Source: Front Microbiol. 2018 Sep 27;9:2206. doi: 10.3389/fmicb.2018.02206 (PMC6171480; doi:10.3389/fmicb.2018.02206)

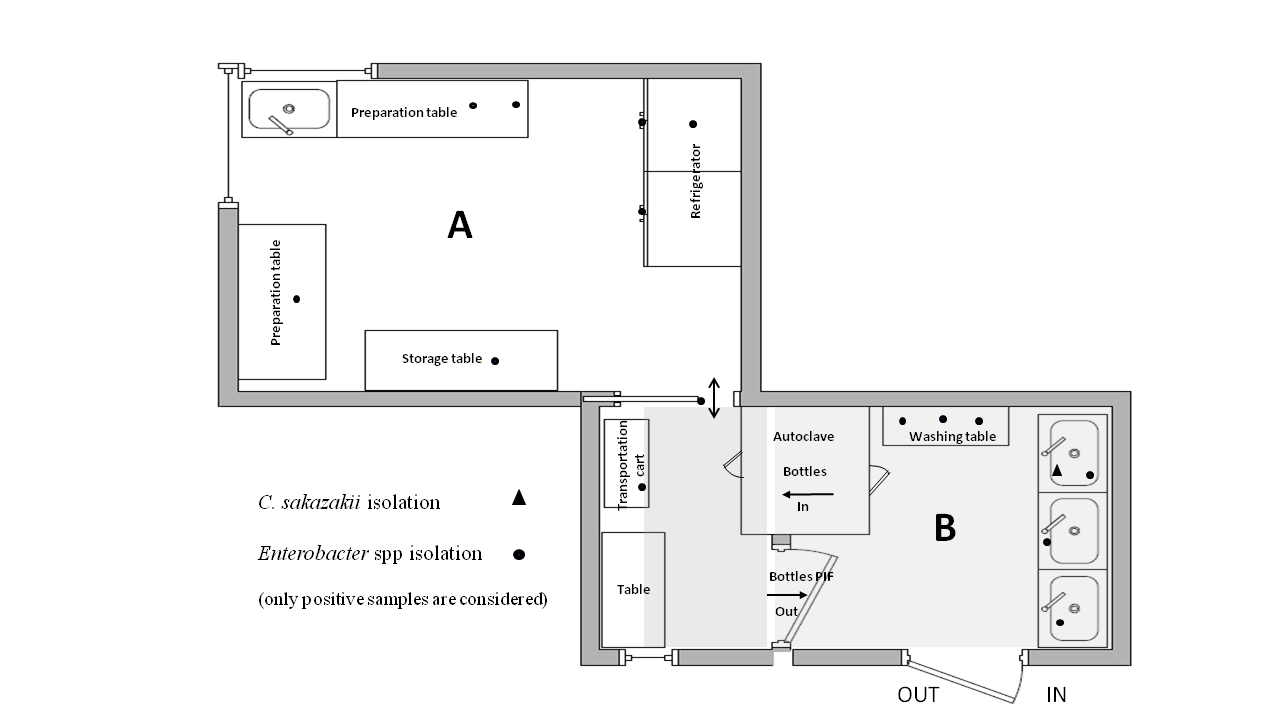

Supplement: FIGURE S1 — Milk kitchen floor plan: (A) PIF bottles preparation area (sterile), and (B) Washing and disinfection area. [file Image_1.tif]
